# Prevalence and factors contributing to missed opportunities for vaccination in Mogadishu, Somalia

**DOI:** 10.1016/j.ijregi.2024.100507

**Published:** 2024-12-03

**Authors:** Abdullahi Mohamed Mohamud, Mohamed Abdirahman Abdi, Abdirahman Mohamed Abdullahi, Abdiweli Mohamed Abdi, Marian Muse Osman, Mohamed Abdelrahman Mohamed, Chukwuma David Umeokonkwo

**Affiliations:** 1 Family Health Department, Federal Ministry of Health, Mogadishu, Somalia; 2 Department of Health Emergencies, Federal Ministry of Health, Mogadishu, Somalia; 3 Department of Public Health, Daffodil International University, Mogadishu, Somalia; 4 Research Department, National Institute of Health, Mogadishu, Somalia; 5 Faculty of Medicine and Health Science, Jamhuriya University of Science and Technology, Mogadishu, Somalia; 6 Faculty of Veterinary Medicine, Somali National University, Mogadishu, Somalia; 7 African Field Epidemiology Network, Kampala, Uganda

**Keywords:** Vaccination, Somalia, Misconception, Vaccine supply

## Abstract

**Objectives:**

Vaccination is a critical public health intervention that significantly reduces morbidity and mortality from vaccine-preventable diseases. Despite the proven benefits of vaccines, missed opportunities for vaccination (MOVs) remain a significant challenge in many low-income countries, including Somalia. This study aimed to determine the prevalence and identify the factors contributing to MOVs in Mogadishu, Somalia.

**Methods:**

A cross-sectional study was conducted at various health care facilities in Mogadishu, Somalia from January to March 2024. A total of 234 caregivers of children under 5 years of age were interviewed using a structured questionnaire. Data on sociodemographic characteristics, health service utilization, and reasons for missed vaccinations were collected. Logistic regression analysis was used to identify factors associated with MOVs.

**Results:**

The prevalence of MOVs in Mogadishu was 26%. The key factors contributing to MOVs included low socioeconomic status, lack of caregiver awareness about vaccination schedules, logistical challenges within the health care system, and misconceptions about vaccine safety and efficacy. Knowledge and beliefs of mothers about vaccination significantly affected the MOVs in children. Not knowing whether all children should be vaccinated is associated with higher probability of (MOVs) (adjusted odds ratio = 13.006, 95% confidence interval = 1.175-143.924, *P* = 0.003).

**Conclusions:**

The study highlighted a significant prevalence of MOVs in Mogadishu, Somalia. Addressing these missed opportunities requires a multifaceted approach, including enhancing public awareness about the importance of vaccination, improving health care infrastructure, and ensuring consistent vaccine supply and proper storage.

## Highlights

• Missed vaccination opportunities in Mogadishu are highly prevalent at 26%.
• Socioeconomic challenges hinder access to timely childhood vaccination.
• Public health infrastructure gaps contribute to vaccination coverage issues.
• Targeted interventions are needed to enhance vaccination rates in Mogadishu.

## Introduction

Vaccination is a cost-effective public health intervention that prevents millions of deaths annually from vaccine-preventable diseases and promotes overall health and well-being [1]. Every year, vaccines prevent around 2.5 million deaths and severe illnesses caused by diseases that can be prevented by vaccination [2]. Nevertheless, despite substantial advancement in global vaccination initiatives, a significant portion of children in low- and middle-income countries still do not receive the vaccines they need, resulting in outbreaks of vaccine-preventable diseases, amplifying child morbidity and mortality rates [3].

In Gavi-supported countries, 71% of children under 12 months old miss DTP1 vaccine recommended by the World Health Organization (WHO), resulting in 12.5 million “zero-dose” children [4]. Health system-related issues, such as missed opportunities for vaccination (MOVs) and inadequate access to health care, contribute to 44% of under-vaccination cases in low- and middle-income countries [5]. MOVs play significant roles in reducing vaccination coverage rates [6].

The WHO defines a MOV as “any contact with health services by an individual who is eligible for vaccination, which does not result in them receiving all the vaccine doses for which he or she is eligible” [7]. Studies have uncovered a high prevalence of MOVs in Africa, with rates ranging from 43% to 57% [8]. Various factors have been documented as contributors to MOVs, these include instances where health care professionals neglect to screen a child's vaccination eligibility during visits for illness, improperly apply false contraindications for vaccination, and exhibit hesitancy in opening multi-dose vaccine vials [9,10].

Somalia, a country grappling with protracted conflict, political instability, and fragile health systems, faces considerable challenges in achieving and sustaining high vaccination coverage rates [11]. Previous studies have highlighted the presence of MOVs in children attending health care facilities where opportunities to administer vaccines are overlooked, regardless of the reason for the facility visit. However, there remains a scarcity of research on the prevalence and factors influencing MOVs in Somalia, particularly, within urban areas such as Mogadishu. Understanding these dynamics is crucial for developing targeted interventions to enhance vaccination coverage, address access barriers, and mitigate the burden of vaccine-preventable diseases in the country.

Therefore, we determined the prevalence of MOVs in children aged 0-59 months attending selected health facilities in Mogadishu, Somalia and identified the contributing factors.

## Material and methods

A MOV was identified when a child eligible for one or more vaccines did not receive the vaccine(s) during a health facility visit. This was measured by reviewing the child's vaccination records and comparing them with the national immunization schedule.

### Study design and setting

A cross-sectional analytical study was undertaken between January and March 2024 across nine purposively selected health facilities in nine districts of Mogadishu, within the Banadir Regional Administration, commonly known as Mogadishu municipality, the capital of Somalia. The estimated population of Mogadishu is 2.6 million of 18 million in Somalia, as per World Meter data based on the latest United Nations figures [12].

In the study area of Mogadishu, Somalia, the national schedule of vaccination services follow the guidelines set by the WHO, which include a series of vaccines administered at specific intervals from birth to 2 years of age, against eight vaccine-preventable diseases: childhood tuberculosis, diphtheria, haemophilus influenza type B, hepatitis B, measles, pertussis, polio, and tetanus, distributed in 607 health facilities in 117 accessible districts of a total of 123 districts, by encouraging families with babies to visit their nearest health facility five times before the children reach 1 year of age. Most of these health facilities, such as mother and child health clinics, are managed by nongovernmental organizations and the Somali Government. Gavi, the Vaccine Alliance, supports all components of immunization activities in 25 accessible districts and provides vaccines and cold-chain equipment in every accessible district [13].

However, several factors impact the adherence to this schedule and the distribution of services. Health care infrastructure is often compromised due to ongoing conflicts and instability, resulting in many health facilities operating with limited resources, including insufficient staffing, irregular vaccine supply, and inadequate storage facilities [14]. Security concerns further restrict access to health facilities for health care providers and the community, with some areas becoming inaccessible due to conflict, thereby reducing the ability of health workers to reach children with routine immunization services [15]. In addition, cultural beliefs and social practices can influence vaccination uptake because mistrust of vaccination programs or misconceptions about vaccine safety and efficacy can lead to resistance or refusal to vaccinate children [16].

Mobile and nomadic populations in Somalia pose a unique challenge for vaccination services because their frequent movement makes it difficult for health workers to ensure that children receive all necessary doses. Economic hardship further impacts families’ access to health care, with transportation and opportunity costs often prohibitive. Various public health initiatives, including outreach programs, mass vaccination campaigns, and community health worker involvement, aim to improve coverage but face inconsistent effectiveness due to these challenges. In addition, variable data reporting and monitoring hinder accurate tracking of vaccination coverage and missed opportunities. Understanding these factors is crucial for interpreting the study's results and improving immunization rates among children under 5 years old in Mogadishu [17].

### Study population and sample size

The study population was mothers or caregivers of children aged 0-59 months and their children who visited nine selected health facilities during the study period. The study included children under the age of 5 years who visited selected health facilities in Mogadishu during the study period. These children needed to be accompanied by their primary caregivers who provided consent for participation. In addition, participants were required to have documented vaccination records for the child through either health facility records or vaccination cards presented by the parent or guardian to accurately assess missed vaccination opportunities. Children without accessible vaccination records and those whose caregivers did not consent to participate were excluded. Children who were critically ill at the time of data collection were also excluded because their immediate health needs took precedence over study participation.

The prevalence of MOVs was derived from a previous study in a similar setting [18]. A study conducted in Wajir County referral hospital reported a prevalence rate of MOVs at 23.2%. This rate was used as a reference to estimate the sample size required to detect prevalence and factors associated with missed vaccinations, with a margin of error of 5% (0.05) and a 95% confidence level (Z = 1.96) using Fischer's formula. Although the calculated sample size was approximately 274, logistical considerations adjusted the sample size to 234 to accommodate resource and feasibility constraints.

### Sampling technique

Health facilities were purposely chosen from various districts within Mogadishu. This selection included public and private health facilities to capture the differences in service delivery and access. Specifically, health facilities offering routine immunization services were targeted to ensure the relevance and accuracy of data collection. Participant selection in the study used a consecutive random probability sampling method, where all mothers or caregivers with children aged under 59 months seeking health care services were identified and interviewed upon completion of their visit. This approach ensured interviews took place after receiving the necessary health care.

### Study tool and data collection

The study used a twofold methodology to evaluate MOVs. The first part comprised the Health Facility Exit Survey for mothers/caregivers who visited the health facility for any reason with a questionnaire adapted from the WHO Essential Programme on Immunization [19] at health care facilities, during which we asked whether the child has ever had vaccinations. Localized modifications were made to ensure relevance and accuracy in the context of Mogadishu, Somalia. This information was assessed with respect to the child's age to determine the presence of any previous MOV. The second component was a desk review of health records and immunization card to verify immunization history and identify any previous missed opportunities. The two data collecting streams provided complementary perspectives regarding the child's immunization status, enabling a more precise evaluation of MOV beyond the particular health care visit, during which the interview took place. The health facility client exit survey (mothers/caregivers) was designed to capture information directly from mothers or caregivers of children aged under 59 months who visited the selected health facilities. The survey aimed to understand their experiences, knowledge, and barriers related to vaccination. Key sections included demographic information (age of the caregiver), relationship to the child (e.g. mother, father, and grandmother), child's vaccination status (age of the child, vaccination history [vaccines received and dates]), reasons for visiting the health facility today, and awareness and attitudes toward vaccination trained research assistants supervised by the principal investigator oversaw data collection.

### Data analysis

Entered data were analyzed using SPSS version 23, with descriptive statistics, tables, and charts to summarize respondent characteristics. A binary logistic regression analysis examined relationships between MOVs and various independent factors, including caregiver demographics and socioeconomic status.

### Ethical consideration

Approval was obtained from the ethical review board of the National Institute of Health (NIH) Somalia (Ref: NIH/IRB/14/DEC/2023), with administrative authorization from selected facilities. Caregivers provided written informed consent before interviews, with assurance of confidentiality and privacy. Children identified as having missed vaccines were referred to nearby facilities for immunization.

## Results

A total of 234 caregivers were interviewed from outpatient and inpatient departments across nine districts in the Banadir region of Somalia. The median age of the caregivers was 26.6 ± 7 years; the majority of caregivers fell into the age group 20-29 years, comprising 74.8% of the total sample. Most caregivers were mothers, accounting for 90.2% of the sample; fathers represented a small proportion (4.3%). Most caregivers were female, constituting 94.5% of the total sample. A significant portion of caregivers had received non-formal education, making up 65.4% of the participants. The majority of caregivers were married; comprising 86.8% of the sample. The majority of caregivers were unemployed; accounting for 80.8% of the sample. According to MOV sociodemographic characteristics of children included in the study, the majority of them were under 6 months old, accounting for 47.4% of the sample. Males accounted up 56% of the total, whereas females made up 44%. The primary reason for the current visit to the health care facility was vaccination, with 66.3% of caregivers bringing their children for this purpose. The majority of deliveries took place in a health facility, accounting for 60.3% of the respondents (Table 1).

**Table 1:** Sociodemographic characteristics of study children and their caregivers at nine selected health facilities in Mogadishu 2024.

Variables	Frequency n = 234	Percentage (%)
**Age of the caregivers (years)**		
<20	12	5.1
20-29	170	72.6
30-39	49	21.0
≥40	3	1.3
**Relationship of caregiver to child**		
Mother	211	90.2
Father	10	4.3
Aunt/Uncle	6	2.6
Grandparent	5	2.1
Siblings	2	0.8
**Gender of caregiver**		
Female	221	94.5
Male	13	5.5
**Education of the caregivers**		
Non-formal	153	65.4
Primary	28	12.0
Secondary	27	11.5
Tertiary	26	11.1
**Marital status of the caregiver**		
Married	203	86.8
Divorced/Separated	20	8.5
Single	8	3.4
Widowed	3	1.3
**Employment status of the caregivers**		
Unemployed	189	80.8
Employed	45	19.2
**Means of transportation**		
Walking	100	42.7
Motorcycle (Bajaj)	62	26.6
Bus	60	25.6
Car	12	5.1
**Age of child (months)**		
<6	111	47.4
6-11	72	30.8
12-23	28	12.0
24-59	23	9.8
**Gender of child**		
Male	131	56.0
Female	103	44.0
**Reason for current visit to facility**		
Vaccination	168	66.3
Medical consultation	58	26.9
Nutrition	12	5.1
Others	4	1.7
**Place of delivery**		
Health facility	141	60.3
Home assisted by trained Midwife	38	16.2
Home assisted by traditional birth attendant	28	12.0
**Vaccination status**		
Vaccinated at least one time	218	93
Never been vaccinated	14	6
Do not know	2	1

**Figure 1:**
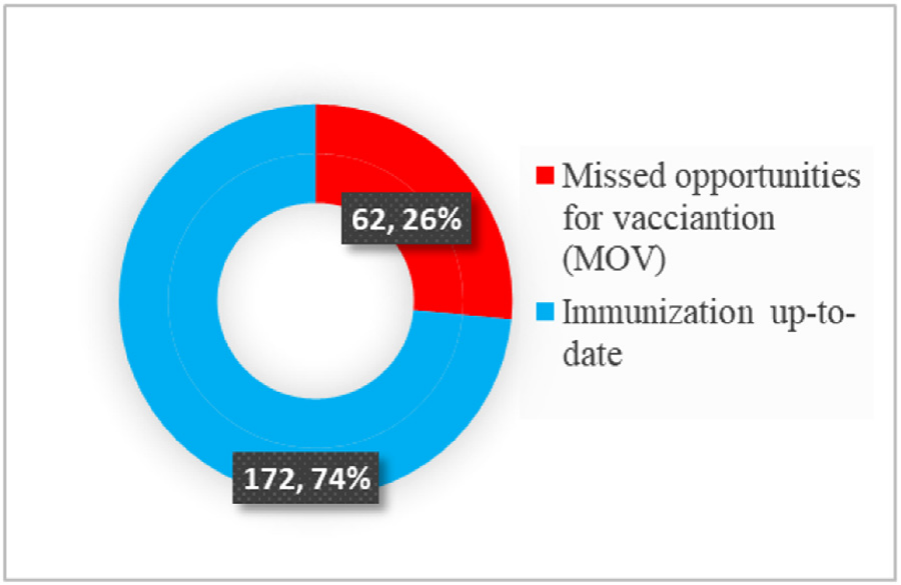
Prevalence of missed opportunities of vaccination at selected health facilities in Mogadishu 2024.

The majority 172 (74%) of children were up to date with their immunizations, whereas a significant portion 62 (26%) of studied children still experienced missed opportunities (Figure 1).

The highest proportion (52%) of MOVs occurred in children younger than 6 months. The primary reasons for not receiving vaccinations were related to issues at health facilities (48%), followed by caregiver-related issues (25, 40%) and a smaller percentage related to health workers (7, 12%) (Table 2).

**Table 2:** Characteristics of children with MOVs (n = 62) at nine selected health facilities in Mogadishu 2024.

Age of child (months)	Frequency	Percentage
<6	32	52.0
6-11	13	21.0
12-23	9	15.0
24-59	8	13.0
**Reason of the current visit for MOVs children**		
General consultation/treatment	33	53.0
Vaccination	16	26.0
Nutrition	11	18.0
Others	2	3.0
**Reasons given for not receiving vaccination**		
Reasons related to the health facility	30	48.0
Reasons related to the caregiver	25	40.0
Reasons related to the health worker	7	12.0

MOV, missed opportunity of vaccination.

The age of the child was significantly associated with MOVs at the bivariate analysis, with younger age groups (aged <12 months) showing significantly decreases the probability of MOVs compared with the reference group (aged 24-59 months) (adjusted odds ratio [AOR] = 0.008, 95% confidence interval [CI] = 0.001-0.072, *P* <0.001). The place of delivery significantly influenced the prevalence of missed opportunity for vaccination; unassisted home deliveries are associated with significantly increased probability of MOVs compared with health facility deliveries (AOR = 6.280, 95% CI = 2.010-19.629, *P* = 0.002). Knowledge and beliefs of mothers about vaccination significantly affected the MOVs in children. Not knowing whether all children should be vaccinated is associated with a higher probability (AOR =13.006, 95% CI = 1.175-143.924, *P* = 0.003), whereas believing that vaccination should not be done is associated with a lower probability than the reference group with (AOR = 0.091, 95% CI = 0.015-0.563, *P* = 0.01). Believing that vaccination cannot have bad effects significantly increases the probability of having unvaccinated children with (AOR = 3.620 95% CI = 1.320-9.924, *P* = 0.01). The reason for visiting the facility does not seem to significantly influence vaccination (all *P* >0.05) (Table 3).

**Table 3:** Multivariable logistic regression output for the factors associated with MOV among children aged 0-59 months at nine selected health facilities in Mogadishu 2024.

Variable	Adjusted odds ratio (95% confidence interval)	*P*-value
**Age group of the child**		
<6 months	0.008 (0.001-0.072)	**<0.001**
6-11 months	0.032 (0.004-0.282)	**0.002**
12-23 months	0.514 (0.084-3.159)	0.472
24-59 months	Ref	Ref
**Place of delivery**		
Home (unassisted)	6.280 (2.010-19.629)	**0.002**
Home assisted by traditional birth attendant	0.685 (0.135-3.466)	0.65
Home assisted by trained midwife	2.994 (0.722-12.410)	0.13
Health facility	Ref	Ref
**Reason for current visit to facility**		
For vaccination	3.087 (0.078-122.004)	0.55
Medical consultation/treatment	0.253 (0.007-9.455)	0.46
Nutrition	0.100 (0.001-12.208)	0.35
Others	Ref	Ref
**Have you heard about vaccination before?**		
Do not know	0.502 (0.072-3.518)	**0.02**
No	0.037 (0.002-0.598)	0.49
Yes	Ref	Ref
**Do you think all children should be vaccinated?**		
Do not know	13.006 (1.175-143.924)	**0.003**
No	0.091 (.015-0.563)	**0.01**
Yes	Ref	Ref
**Do you think vaccination can have bad effects on your child's health?**		
Do not know	1.187 (0.271-5.204)	0.82
No	3.620 (1.320-9.924)	**0.01**
Yes	Ref	Ref

Ref, reference.

## Discussion

Immunization is the most successful and effective health strategy for preventing and eradicating childhood illness worldwide. In Somalia, it is a big challenge to achieve and maintain high immunization coverage in all regions due to inaccessibility, lack of strong health system, displacement, and droughts [17].

The analysis revealed that 26.5% of the children had MOVs and did not complete all the vaccine doses for which they are eligible, whereas 6% were zero-dose children and had never been vaccinated. These rates have a significant negative impact on achieving the required vaccine coverage in the country.

In addition, we evaluated the factors significantly associated with MOVs, including child's age, place of delivery, non-vaccination visits, and lack of vaccine information, and caregiver's knowledge and attitude toward vaccines.

The prevalence of MOVs in Somalia is similar or close to the prevalence rates found in other African countries. The study also compared the coverage of immunization in the studied population (73.5%) with the national coverage (30.40%) and found a higher coverage, attributed to the accessibility and availability of services in the Banadir region [20]. Conducting further studies at the national level to gain a comprehensive and representable understanding of missed opportunities for vaccination in Somalia is highly recommended.

The major reason of MOVs was due to the health facility–related (stock out, not opening vaccine vial for one child, unclear working hours which was approximately 48%), followed by reasons related to the caregiver (40%) and reasons related to the health worker [13]. The mean time that caregivers waited to vaccinate their children was 10.82 ± 12.95 minutes.

Similar studies conducted in Mozambique and in Maasai nomadic pastoralists in Kenya, 25.7% and 30.1%, respectively, identified high prevalence rates of MOVs. Factors such as accessibility to vaccination sites, lack of schooling for mothers, and children born at home or outside the country were associated with incomplete vaccination. The prevalence of missed opportunities in the Maasai nomadic pastoralist study was higher than in the Somali study due to differences in the target population (rural vs urban). Geographic mobility and distance to the health facility were also key determinants of severe under-vaccination in pastoralists in Kenya [21,22]. Developing strategies for reaching the children living in rural and inaccessible areas can reduce the MOVs. This may involve mobile vaccination clinics, community health workers, and volunteers to overcome geographical barriers and ensure equitable access to immunization service.

Our study revealed that among the missed children, the percentage with MOVs was significantly higher in those aged <6 months (51.6%), unlike in a similar study conducted in Burkina Faso in 2020 that showed that the percentage with MOVs was significantly higher in those aged >12 months [10]. This discrepancy could be due to differences in the nature of the study, geographics, health infrastructure, and the study design.

The result of multiple binary logistic regression analysis shows that children who were delivered in the home unassisted (AOR = 6.280, 95% CI: 2.010-19.629) were six times more likely to be unvaccinated than their counterparts. The findings were consistent with a community-based study in the Nairobi province (Kibera Division, Embakasi Division, and West-lands Division) that revealed that 23.8% was the overall prevalence of missed opportunity for immunization in the study population [23]. This could be due to similarities in the nature of the study, geography, and the study design.

Regarding the caregivers knowledge and attitude, this study was consistent with another population-based cross-sectional study conducted in East Pokot, Baringo County, Kenya, with a prevalence of 23%, which reported that the number of children in the family, the location of the child's birth, literacy level, knowledge of the recommended immunization schedule, a nomadic lifestyle, the distance to the nearest medical facility, and the area of residence (urban/rural) were all factors that contributed to low immunization coverage [24].

Interventions should be tailored to address sociodemographic factors influencing vaccination rates, such as place of delivery. Strategies may include promoting institutional deliveries, providing targeted support and education for caregivers who opt for home births, and ensuring access to vaccination services in both urban and rural areas.

The government and the partners should implement the 10-step process of the MOV strategy. This evidence-based approach includes identifying missed opportunities, training health care providers, improving data collection systems using an electronic system, and implementing reminder systems to reduce missed vaccination opportunities.

Regarding the knowledge of the caregivers, caregivers who do not know whether the child should be vaccinated (AOR = 13.006, 95% CI: 1.175-143.924) is 13 times more likely to have missed opportunities for immunization than the caregivers who know.

The proportion of MOVs was associated with caregiver attitude; the multiple binary logistic regression analysis result showed that the participants who have no idea about the side effects of vaccines (AOR = 3.620, 95% CI: 1.320-9.924) were three times more likely to miss the vaccination they are eligible to receive than the participants who know the side effects. The possible explanation is that caregivers who are concerned about potential vaccine side effects may choose to refuse or delay immunizations. Conversely, caregivers who are unaware of the importance of vaccination or uncertain about whether their child should be vaccinated may fail to attend immunization sessions.

## Conclusion

The study highlighted that MOV is a significant public health challenge in Mogadishu, Somalia, with a notable prevalence of missed vaccination opportunities. Key contributing factors include child's age, place of delivery, non-vaccination visits, and caregiver's knowledge and attitude toward vaccines. Addressing these requires a multifaceted approach: increasing public awareness, improving health care infrastructure, ensuring reliable vaccine supply chains, and implementing tailored community engagement and educational campaigns to overcome misconceptions and build trust in vaccination programs.

## Declarations of competing interest

The authors declare that they have no known competing financial interests or personal relationships that could have appeared to influence the work reported in this paper.
